# Hospitalization and Socio-Health Care for Dementia in Spain

**DOI:** 10.3390/jcm9123875

**Published:** 2020-11-28

**Authors:** Carlos Llanes-Álvarez, Jesús M. Andrés-de llano, Ana I. Álvarez-Navares, Carlos Roncero, M. Teresa Pastor-Hidalgo, José R. Garmendia-Leiza, Irene Andrés-Alberola, Manuel A. Franco-Martín

**Affiliations:** 1 Department of Psychiatry, Complejo Asistencial de Zamora, 49022 Zamora, Spain; mfrancom@saludcastillayleon.es; 2 Department of Pediatrics, Complejo Asistencial Universitario de Palencia, 34005 Palencia, Spain; jandresl@saludcastillayleon.es; 3 Department of Psychiatry, University of Salamanca Health Care Complex, 37007 Salamanca, Spain; aialvarez@saludcastillayleon.es (A.I.Á.-N.); croncero@saludcastillayleon.es (C.R.); 4 Castilla y León Health Authority, Complejo Asistencial de Zamora, 49022 Zamora, Spain; mtpastor@saludcastillayleon.es; 5 General Direction of Information Systems, Quality and Pharmaceutical Provision at Castilla y León Health Authority, Regional Health Management, 47007 Valladolid, Spain; jgarmendia@saludcastillayleon.es; 6 Castilla y León Health Authority, Complejo Asistencial Universitario de Palencia, 34005 Palencia, Spain; iandresal@saludcastillayleon.es

**Keywords:** dementia, cognitive dysfunction, hospitalization, trends, big data, health information system, psychosocial support, Spain

## Abstract

Dementias are brain diseases that affect long-term cognitive and behavioral functions and cause a decrease in the ability to think and remember that is severe enough to disturb daily functioning. In Spain, the number of people suffering from dementia is rising due to population ageing. Reducing admissions, many of them avoidable, would be advantageous for patients and care-providers. Understanding the correlation of admission of people with dementia and its trends in hospitalization would help us to understand the factors leading to admission. We conducted a cross-sectional study of the hospital discharge database of Castilla y León from 2005 to 2015, selecting hospitalizations for dementia. Trends in hospitalizations by year and age quartiles were studied by joinpoint regression analysis. 2807 out of 2,717,192 total hospitalizations (0.10%) were due to dementias; the main groups were degenerative dementia (1907) followed by vascular dementia (607). Dementias are not a major cause of hospitalization, but the average stay and cost are high, and many of them seem avoidable. Decreasing trends were detected in hospitalization rates for all dementias except for the group of mild cognitive impairment, which grew. An increasing–decreasing joinpoint detected in 2007 for vascular dementia and the general downward hospitalization trends for most dementias suggest that socio-health measures established since 2007 in Spain might play a key role in reducing hospitalizations.

## 1. Introduction

Dementia is associated with increased risk of all-cause hospitalizations [1]; these admissions are costlier for people with dementia than for those without [2], and many of them are treatable on an ambulatory basis [3]. The prevalence of dementia is approximately 5% among individuals older than 65 years old, but it increases up to 20–40% among individuals older than 85 years [4]. The number of older people and older people with dementia appears to increase, with a current mean prevalence of dementia reaching 9.33 ± 8.21% [5,6], probably due to the increased life expectancy and aging of western societies [7]. Degenerative dementias, especially Alzheimer’s disease, are the most common ones (up to 60–70% of all cases). They are followed by vascular dementias, mainly caused by stroke, accounting for about 25% of dementia cases [8]. Induced dementias are a broad group of types of non-specific softening of the brain, among which stands out mental deterioration due to drugs and alcohol; it is also known as “alcohol dementia” because of the preponderance of this substance [9]. Finally, mild cognitive impairment gathers those who are between the expected cognitive decline of normal ageing and the more serious decline of dementia [10]. Despite this, the clinical reality is often different, and we frequently find a mixture of dementias: degenerative, vascular, induced, or mild cognitive impairment.

The treatment of dementia is determined by its cause. The therapy for alcoholism and drug addiction might prevent the development of some induced dementias. The control and treatment of cardiovascular risk factors and diseases is commonly accepted as the main treatment for vascular dementia. As currently there are no disease-modifying treatments for any of the neurodegenerative dementias, clinicians try to mitigate its cognitive and behavioral effects through a broad and diverse range of limited-effectiveness therapeutic tools to minimize harm to patients with dementia [11,12].

Dementia is the main cause of dependence in Spain and in most developed countries, and it psychologically, physically, socially, and economically affects the patients and their entire families [13]. Hospitalizations account for almost half of total healthcare costs [14], and even more in demented patients [15]. The reduction of hospitalizations might improve the quality of life of these patients and their families, and it might lead to cost savings in the expenses of dementia care, as many admissions are avoidable by replacing them with an optimal ambulatory care plan [1].

If we assume that there are no disease-modifying treatments for any of the neurodegenerative dementias, the limited efficacy of individual therapeutic interventions (even combined), and the social scale of the problem, it is easy to understand why governments have focused on social-health care for dementias [12]. Social support, a social network’s provision of psychological and material resources to benefit an individual’s ability to cope with stress, may serve as an enabling resource for health services use. Social support may buffer against the likelihood of hospitalization. In Spain up until 2007, care services for dependent persons were imbalanced. On the public side, such care depended on social security, public health services, and many other dispersed social services. However, it also depended on the initiative of families and charities. On the 1 January 2007, the 39/2006 law, “Promoción de la Autonomía Personal y Atención a las Personas en Situación de Dependencia de España” (translated as: Personal Autonomy and Dependent Care Law), came into effect in Spain. It guarantees public support for people who cannot lead independent lives due to illness, disability, or age. The aim of healthcare integration is to move from a fragmented framework, in which individuals apply for and receive health benefits and care benefits separately, to a new model of shared responsibility; the law establishes a catalogue of services that dependent people can enjoy. Many types of services and benefits are provided: prevention services, telecare, home help, day-care centers, and residential care services. Exceptionally, the beneficiary may receive an economic benefit to be cared for by non-professional (usually relatives) caregivers, as long as the right conditions exist.

To assess the impact of social policies on health outcomes is always a tricky question, even when some kind of relationship might exist, and making associations between health outcomes and non-health issues is a sensitive matter. Hospitalizations are one of the most objective and reliable indicators, and the analysis of large data sets to establish general trends over the time is the best approach to an issue like this [16]. In Spain, the Conjunto Mínimo Básico de Datos (translated as: “Basic Minimum Data Set” and hereinafter referred to as CMBD) is the broadest administrative clinical database, and its fulfillment is mandatory in public hospitals [17]. The aim of our research is to describe the hospitalization for dementia in Castilla y León between 2005 and 2015. In this Spanish region, dependency is synonymous with dementia due to ageing of its population. Castilla y León is the biggest region not only in Spain but in Europe too, and it is also recognized for an exemplary application of the law of dependency. For all this, it is an optimal sample to discuss the impact of social measures on health outcomes like hospitalization; the mutual understanding of how society and health interact may contribute to the improvement of both areas.

## 2. Experimental Section

We conducted a cross-sectional research of the hospital discharge database of Castilla y León from 2005 to 2015, selecting hospitalizations with a dementia diagnosis at discharge. We distinguished between the four most common clinical types of dementia according to the following ICD-9 MC codes (Table 1). Trends in the rates of hospitalization/1000 hospitalizations per year and differences according to age-quartile groups were studied by joinpoint regression analysis.

Sample: The data came from the CMBD, which provides important information about the health reality of a population, since in addition to collecting usual demographic data (age, sex, urban or rural residence), the CMBD records the diagnosis that motivated hospital admission (main diagnosis). Finally, the CMBD includes the patient’s date of admission and discharge, as well as the circumstance of admission (urgent or scheduled) and circumstance of discharge (discharge to their home, death, transfer to another hospital, etc.). The coding in the CMBD is done based on hospital discharge reports, and since they correlate closely with hospital admission, we use both terms synonymously. The target population in Castilla y León in the middle of the study period (Instituto Nacional de Estadística 2010) was 2,547,408 people [18]. For the standardization of rates by age, the European standard population of 2013 was used.

The study population is made up of 2807 cases with a main diagnosis of dementia discharged from the public hospitals of Sanidad de Castilla y León (SACYL) between 2005 and 2015. Individuals were classified based on the criteria of the CMBD and the International Classification of Diseases, Ninth Revision, Clinical Modification (ICD-9-MC). The data that support the findings of this research are available at the Dirección General de Sistemas de Información, Calidad y Prestación Farmacéutica, located at: Pseo. de Zorrilla, 1. C.P.: 47007 Valladolid (Spain). Legal restrictions apply to access these data (Ley 16/2003, de 28 de mayo, de cohesion y calidad del Sistema Nacional de Salud), which required the data be acquired with the relevant authorization in this study. We provide a link to the access conditions: https://www.boe.es/eli/es/l/2003/05/28/16.

Variables: the study variable was discharges from hospitals in Castilla y León between 2005 and 2015. Hospitalizations with a primary and secondary dementia diagnosis at discharge were selected. Main diagnoses for hospital discharges according to selection of ICD-9-MC codes were used in previous research [19]. Codes used are detailed in Table 1, and the groups selected were degenerative, vascular, and induced dementia and mild cognitive impairment. Statistical and trend analysis: general descriptive data for the whole group and for each dementia studied were derived. Incidence rates were calculated per 1000 hospitalizations per year, global and specific, by type of dementia, and the trend over the 11 years was studied, in general and by groups. Analysis of trends to determine changes in rates with significant statistical differences over time was performed by linear joinpoint regression, a test that assesses the trend over time in years and age quartiles for the series of selected patients. The joinpoint regression model was created by the National Cancer Institute (NCI) to asses cancer mortality rates. It has been identified as a valuable tool for making inferences about changes in trends over time, and it has been used in numerous domains to assess changes in time series data. Joinpoint fits the selected trend data (hospitalization rates) into the simplest joinpoint model that the data allow. In this analysis, the points of change (joinpoints or inflection points) show statistically significant changes in the trend (ascending or descending). Graphically, the joinpoint models performed on the logarithm of the rate describe a sequence of connected segments. The point at which these segments come together is a joinpoint and represents a statistically significant change in the trend. In addition, for each segment, an annual change percentage was calculated for each trend by means of generalized linear models, assuming a Poisson distribution and showing in each case its level of associated statistical significance; the input data to this analysis are the hospitalization rates adjusted by age to the European Standard Population (Publications Office of the European Union 2013) with 95% confidence intervals (95% CI). We used preset parameters of the version 4.8.0.0 of joinpoint free access software from the Research and Surveillance Program of the National Cancer Institute of the United States. Values of *p* < 0.05 were considered statistically significant differences.

To check if the results obtained for trends in each group of dementia were replicated, we have done an age-quartile analysis in a larger sample, which includes all the hospitalizations for dementia along the period of research. Quartile analysis divides the data sample into four equal parts. It might also be useful to assess the spread and central tendency of the data and to see the evolution of hospitalization and how socio-health measures may impact on it according to the age.

Ethical approval: This research was conducted according to the highest ethical standards and methods for the conduct of quality human biological rhythm research [20] and was approved by the ethical committee of the Zamora Health Care Complex.

## 3. Results

Healthcare in Spain consists of both private and public healthcare; about 90% of Spanish people use the public healthcare system, which is called the National Health System. However, it is very decentralized, with service delivery organized at regional level according to its specific needs. Castilla y León comprises 14 public hospital centers in nine provinces. The CMBD of hospital discharges from Castilla y León, between 2005 and 2015, consists of 2,717,192 records. A total of 2807 (0.1%) of them were hospitalizations for dementia in all departments in public centers in Castilla y León. All dementia diagnosis were grouped into four clinical categories and selected according to the indicated codes (Table 1). Degenerative dementia was the main diagnosis with 1957 hospitalizations (69.70%), followed by vascular dementia with 607 hospitalizations (21.6%), induced dementia with 89 hospitalizations (3.17%), and finally mild cognitive impairment with 154 hospitalizations (5.49%). A total of 2807 hospitalizations for any type of dementia were analyzed by frequency, percentage, and average age (Table 2) and for department of hospitalization (Table 3). Results are shown in Table 2, Table 3, and Table 4. In this sample, 46.2% were male versus 53.8% female; 1535 cases (54.7% of the sample) came from urban areas and 1248 (44.5%) from rural areas. 2641 (92.4%) cases were urgent hospitalizations and just 166 scheduled. With 126 deaths, the in-hospital mortality was 4.5%.

Case mix index (CMI) is a relative value assigned to a diagnosis-related group of patients in a medical care environment. The CMI value is used to determine the allocation of resources to care for and/or treat patients in the group. Admissions are classified into groups having the same condition (based on main and secondary diagnosis, procedures, age), complexity (comorbidity), and needs. These groups are known as Diagnosis Related Groups (DRG) or Resource Use Groups (RUG). All the groups showed similar CMI values (average: 1.0368); DRG pricing varies in terms of geographic variation (labor markets, etc.), and it varied between 4609€ for degenerative dementia and 5301€ for the rest. A finding may be statistically significant; nevertheless, it may lack clinical or healthcare significance, as is our view in this case. We provide an aggregate analysis of health economics outcomes between degenerative and non-degenerative dementias (Table 2). By department, 55.5% of hospitalizations corresponded to Internal Medicine, and the rest were attributable to neuropsychiatric symptoms (Table 3). We provide demographics, and a novel health indicator as the ratio of hospitalization for dementia adjusted to age-structure by Spain demographics (Table 4).

**Figure 1:**
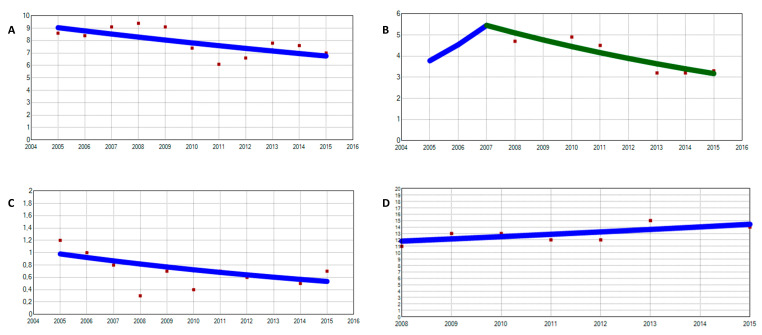
Trends in hospitalization for dementia. Hospitalization rates for dementia per 100,000 inhabitants. Analysis by groups of the dementias studied (**A**–**D**, from top to bottom) with inflection points (joinpoints) and APC. (**A**) Degenerative dementia: 0 joinpoints, APC 2005–2015 −2.9 (95% CI −5.5 to −0.1, *p* < 0.01 *). (**B**) Vascular dementia, 1 joinpoint (2007) APC 2005–2007 20.1 (95% CI −11.8 to 63.6, *p* = 0.2), APC 2007–2015 −6.5 (95% CI −9.5 to −3.5 *p* = 0.01 *). (**C**) Induced dementia, 0 joinpoints, APC 2005–2015 −5.9 (95% CI −10.6 to −0.9, *p* < 0.01 *). (**D**) Mild cognitive impairment, 0 joinpoints, APC 2008–2015 2.9 (95% CI −0.1 to 6, *p* = 0.01 *). APC: annual percentage change; 95% CI: 95% confidence interval. (*): APC statistically significant. Red dots: exact annual value. Lines represent trends, with line colors changing where joinpoints were identified. A blue line only represents a monotonic trend. *X*-axis: years (from 2004 to 2016). *Y*-axis: discharge rate for dementia group or quartile studied; discharge rates for dementia/1000 hospital discharges. APC: annual percentage of change. 95% CI: 95% confidence interval. Data represent exact annual value. * Statistically significant CAP. Note: some exact annual values (red dots) might be overlapped by the regression line in some graphs (see graph **B** -2005, 2006, and 2007- or graph **D** 2014).

**Figure 2:**
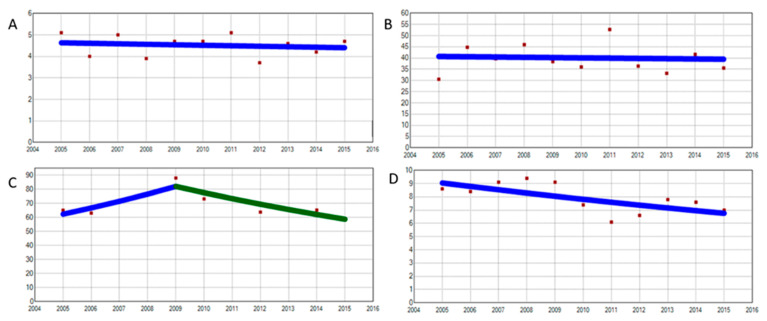
Trends in psychiatric hospitalization by age quartiles. (**A**) First quartile, 0 joinpoint, APC 2005–2010 −0.5 (95% CI −2.9 to 2, *p* = 0.7). (**B**) Second quartile, 0 joinpoints, APC 2005–2015 −0.3 (95% CI −4.1 to 3.6, *p* = 0.9). (**C**) Third quartile, 1 joinpoint, APC 2005–2009 7.1 (95% CI 1.8 to 12.7 *p* < 0.01) APC 2009–2015 −5.4 (95% CI −7.9 to −2.9; *p* < 0.01). (**D**) Fourth quartile, 0 joinpoints, APC 2005–20015 −2.9 (95% CI −5.5 to −0.1, *p* < 0.01 *). APC: annual percentage change; 95% CI: 95% confidence interval. (*): APC statistically significant. Red dots: exact annual value. Lines represent trends, with line colors changing where joinpoints were identified. A blue line only represents a monotonic trend. *X*-axis: years (from 2004 to 2016). *Y*-axis: discharge rate for each dementia studied; discharge rates for drugs/1000 hospital discharges. APC: annual percentage of change. 95% CI: 95% confidence interval. Data represent exact annual value. * Statistically significant CAP. Note: some exact annual values (red dots) might be overlapped by the regression line in some graphs (see graph **C** 2007 and 2008).

The general trends in annual hospitalization for dementia were down and statistically significant: −2.9% (*p* < 0.01) for degenerative, −6.5% (*p* = 0.01) for vascular -between 2007 and 2015-, and −5.9% (*p* < 0.01) for induced dementia. This is a surprising finding for an arising pathology that we will discuss later. Vascular dementia showed a joinpoint in 2007, describing an increasing–decreasing graph. The detection of such a +20.1% to −6.5% annual trend change is always a disturbing finding. Nonetheless, changes in coding or organizational changes such as the closure of hospital beds have not been found, so we assume it is accurate and valid. On the other hand, mild cognitive impairment displayed a +2.9% annual percentage of upward trend, probably due to an increase in the reporting of this clinical entity (Figure 1). The trend analysis by age quartiles showed no clear tendencies for the first and second quartiles and two downward annual trends (*p* = 0.01 *) of −5.4 and −2.9%, respectively. Age quartiles distribution was 1st quartile: 0–34 years old; 2nd quartile: 34–45; 3rd quartile 45–58; and 4th quartile 58 years and older (Figure 2).

## 4. Discussion

Just estimations or small and partial studies, but no actual data on the prevalence of hospitalization for dementia, are available in Spain [21,22,23]. According to previous research, the prevalence of the disease is around 0.05% among people aged 40 to 65, 1.07% between 65–69 years, 3.4% between 70–74 years, 6.9% between 75–79 years, 12.1% between 80–84 years, 20.1% between 85-89 years, and 39.2% among those over 90 years old. If we consider the Castilla y León’s INE population data (2015) and apply it to these prevalences, the number of people affected in this region exceeds 37,000 people between those over 40 years old with dementia [24], and so on. Our annual rate of hospitalization for these patients is about 0.7/person-year, in line with the hospitalization rates in people with dementia found by high-quality studies, which were between 0.37 and 1.26/person-year. In our sample, 1375 (70%) of all dementia hospitalizations correspond to degenerative dementias, mostly Alzheimer’s disease; this is also in accordance with prior findings [15]. The average age is 70.78 ± 9.7 years (Table 2). The length of stay is 12.7 days, 49% higher than the average hospital stay in the region (8.48 days), but much lower than, for example, the average of 18.5 days of hospitalization due to psychiatric illnesses [25]. In a disaggregated analysis between degenerative dementia and the rest of them (vascular, induced, and mild cognitive impairment), we found that the average stay is slightly superior (13.09 vs. 12.33), but AP27-DRG weight and cost were statistically significantly lower *p* < 0.001 for degenerative dementia than the rest; this is most probably due to the high physical comorbidity associated to the non-degenerative dementia group.

The trend analysis showed a statistically and significant downward trend for degenerative and induced dementias of −2.9% and −5.9% annual reduction, respectively. Vascular dementia exhibited an up-and-down figure with a joinpoint in 2007 that started a -6.5% annual downward trend after a 2005–2007 biennium with an upward trend of 20% per year. Apart from this peak, difficult to explain, the downward trends in hospitalization for dementia are the norm in this sample along the research period; however, 2007’s peak was an inflection point that matched up with an important event: the implementation of Law 39/2006, of December 14th, “Promoción de la Autonomía Personal y Atención a las personas en situación de dependencia de España”, better known as the “dependency law”. This is the Spanish law that created the current System for Autonomy and Dependency, a set of services and benefits aimed to promoting personal autonomy through accredited, concerted public and private services (Table 5) [15]. These results were similar to the ones obtained in the age-quartile analysis and seem to support them. However, the up-and-down figure with a peak in 2009 observed in vascular dementias and in the 3rd quartile is remarkable, because of the “youth” (45–58 years old) of the patients included in it. Our hypothesis is that vascular dementia is the one with the earliest onset and the worst behavioral disturbances, and so on the impact of social health benefits in the reduction of hospitalizations is higher. Readers might wonder under what circumstances a M.C.I. patient was admitted if secondary diagnosis was discarded and the clinical mildness of this condition does not usually precipitate admission. Probably part of these admissions were due to another reason; however, the doctor who did the coding at the discharge considered that this admission was due to M.C.I.; as an example, a hypoglycemia due to an accidental self-administered insulin overdose in a M.C.I. patient would be one of these admissions in which the root cause would always be M.C.I. To assess the impact of social measures on health outcomes is a thorny issue, a leap in the dark, as there are no specific indicators for the evaluation of both. This research provides a big-data approach to establish long-term hospitalization trends for dementia looking for patterns and correlations, which may prompt unexpected insights to optimize hospitalization, improve dementia care, and even shape the law’s future. Castilla y León is the best region in Spain to carry out this research for several reasons: it is the largest region of Spain and Europe; its population, very old, is distributed in a balanced way between rural and urban areas; and it is a leader in the management of dependency with a Spanish Observatory score for Dependency of 9.29 out of 10 compared to 4.86 for the Spanish average [18].

From the Department of Hospitalization, we found how Internal Medicine with 1559 hospitalizations accounted for the 55.5% of the whole dementia hospitalization. These admissions probably correspond to organic pathologies in patients diagnosed with dementia; they are difficult to avoid and not of our main concern in this research; however, we took notice that the remaining 44.5% of hospitalizations end up in Neurology (413 -14.7%-) and Psychiatry (835 -29.7%-). The authors think that the initial diagnostic study and behavioral disturbances are the cause of these admissions, which are otherwise potentially avoidable. For instance, the old age psychiatrists (OAP) of the National Health System (NHS) in U.K. are organized in psycho-geriatric units with their own dedicated ward, where patients are treated by super-specialist geriatric psychiatrists, psychologists, and nurses. This model has shown to be effective in reducing hospitalizations [26]. In our view, this is the best holistic, integrated approach to the legal, social, physical, and mental health problems in old age. A pilot program of a psychogeriatric unit, inspired by the NHS, has been carried out in Zamora since 2010. This province has the highest rate of people aged 65 years or older (30.2%) and, in the absence of a specific assessment, its hospitalization rates appear to be average rather than at the top (Table 4). The increasing frequency of dementia and a high average stay and cost (12.7 days and 4962.4€ -GRD-AP 27 (2014)) seem to support the cost effectiveness of the widespread implementation of these outpatient consultations. Looking for differences between the management of dependency in Castilla y León and the whole country, it looks like the imbalance towards the benefits linked to the service (78% of all benefits granted) vs. the national average of 66.1% may have some impact on the hospitalization.

Limitations: we recognize underreporting in the coding of dementia at discharge might be the main limitation of this research. Furthermore, the data were obtained retrospectively from a non-specifically clinical administrative record; despite this, we have checked that changes in coding have not been undergone over the years and in the different hospitals. Nevertheless, the analysis of databases as the CMBD, with a large volume of information, is one of the best approaches to the reality of a pathology.

## 5. Conclusions

Hospitalization is frequent in people suffering from dementia (7.5%), although dementia remains a minor cause of admission to the general hospital (0.1%). Despite the prevalence of dementia, it does not stop growing, due mainly to the aging of the population; there is a surprising downward trend in hospitalizations for dementia. The Spanish Dependency Law (39/2006) seems to have changed tendencies or consolidated previously established downward trends; Castilla y León has provided more benefits linked to professional services than to family care, which might become a reference for the future benefits allocation elsewhere.

## Figures and Tables

**Table 1:** Main diagnoses for hospital discharges and associated ICD-9-MC codes.

	Diagnosis Group	ICD-9-MC
I	Degenerative dementias	290.0–290.3, 331.0–331.1, 331.11, 331.19, 331.82
II	Vascular dementias	290.40–290.43, 294.20–294.21
III	Induced dementia	291.2, 292.82
IV	Mild cognitive impairment	331.83

The sample was classified into three diagnoses groups of dementia (degenerative, vascular, and induced) and mild cognitive impairment (left) according to the following International Classification of Diseases, Ninth Revision, Clinical Modification (ICD-9-MC) codes (right).

**Table 2:** Healthcare and economic assessment of the sample.

		*n*	Average	SD	*p*
Age (years)	Vascular/Induced/M.C.I	1432	79.0	10.5	0.2
	Degenerative Dementia	1375	78.5	8.8	
	Total	2807	78.8	9.7	
Length of stay (days)	Vascular/Induced/M.C.I	1432	12.3	13	0.2
	Degenerative Dementia	1374	13.1	17.3	
	Total	2806	12.7	15.3	
AP27-DRG Weight (2014)	Vascular/Induced/M.C.I	1432	1.11	0.52	<0.001
	Degenerative Dementia	1375	0.96	0.43	
	Total	2807	1.04	0.48	
Cost AP27-DRG (2014)	Vascular/Induced/M.C.I	1432	5301.4€	2484.5	<0.001
	Degenerative Dementia	1375	4609.3€	2070.9	
	Total	2807	4962.4€	2316.8	

Number of hospitalizations and average ± SD -standard deviation- of the age, length of stay, and AP27-DRG weight and cost (in euros) classified in degenerative dementias and a non-degenerative dementias group that includes mild cognitive impairment and induced and vascular dementia.

**Table 3:** Department of hospitalization by diagnosis group.

			Diagnosis Group				Total
			Degenerative Dementia	Vascular Dementia	Induced Dementia	M. Cognitive Impairment	
Department of hospitalization	Internal Medicine	Cases	1029	405	54	71	1559
		Department of hospitalization (%)	66.0%	26.0%	3.5%	46%	100%
		Diagnosis group (%)	52.6%	66.7%	60.7%	46.1%	55.5%
	Neurology	Cases	290	69	8	46	413
		Department of hospitalization (%)	70.2%	16.7%	1.9%	11.1%	100%
		Diagnosis group (%)	14.8%	11.4%	9.0%	29.9%	14.7%
	Psychiatry	Cases	638	133	27	37	835
		Department of hospitalization (%)	76.4%	15.9%	3.2%	4.4%	100%
		Diagnosis group (%)	32.6%	21.9%	30.3%	24.0%	29.7%
Total		Cases	1957	607	89	154	2807
		Department of hospitalization (%)	69.7%	21.6%	3.2%	5.5%	100%
		Diagnosis group (%)	100%	100%	100%	100%	100%

Hospitalizations and its percentages by department of hospitalization (internal medicine, neurology, or psychiatry) and diagnosis group (mild cognitive impairment, degenerative, vascular, and induced dementia).

**Table 4:** Socio-health care benefits for dementia in Castilla y León and hospitalization rates for dementia.

	Total of Care Home Vacancies (a)	Total Population (b)	People Aged 65+ Years (c)	Ratio Home Care/People 65+ Years (a/c*100) (d)	% People Aged 65+ Years (c/b*100)	Hospitalizations for Dementia		Ratio (*n*/d)
						*n*	%	
Ávila	3525	160,700	41,034	8.6	25.5	147	5.2%	17.1
Burgos	6656	358,171	82,714	8.0	23.1	430	15.3%	53.75
León	7117	468,316	123,508	5.8	26.4	622	22.2%	107.24
Palencia	4297	163,390	40,138	10.7	24.6	320	11.4%	29.9
Salamanca	6915	333,603	86,961	8.0	26.1	192	6.8%	24
Segovia	2962	154,184	34,538	8.6	22.4	146	5.2%	16.97
Soria	2486	88,903	22,705	10.9	25.5	173	6.2%	15.87
Valladolid	7271	521,130	115,122	6.3	22.1	389	13.9%	61.74
Zamora	4336	177,404	53,554	8.1	30.2	366	13.0%	45.18
Castilla y León	45,565	2,425,801	600,274	7.6	24.7	2807	99.2	369.3
Spain	366,633	46,572,132	6,764,204	4.2	18.8	No data	No data	No data

Total of care home vacancies, total population, people aged 65 or older, and ratio of care home vacancies (data from the Spanish Observatory for Dependency). Hospitalizations for dementia and its ratio adjusted by age for each one of the nine Castilla y León provinces (data from original research findings).

**Table 5:** Distribution by kind of social health benefit and percentage of total benefits granted in Castilla y León and Spain.

Benefits	Total Benefits Granted in Castilla y León	Benefits Granted (% Castilla y León)	Benefits Granted (% Spain)
Prevention dependence and personal promotion	12,500	11.57%	3.67%
Telecare	8320	7.70%	15.56%
Home help	22,274	20.62%	15.95%
Day-care centers	8276	7.66%	8.02%
Residential Care	8286	7.67%	13.88%
BENEFITS LINKED TO THE SERVICE	24,315	22.51%	8.42%
Personal assistance benefits	321	0.30%	0.56%
SUBTOTAL SERVICES	84,292	78.02%	66.07%
Family care benefits	23,744	21.98%	33.93%
SUBTOTAL BENEFITS ECONOMIC	23,744	21.98%	33.93%
Total benefits	108,036	100%	100%

The *Personal Autonomy and Dependent Care Law (39/2006)* guarantees dependent people access to different benefits. In broad terms, the government finances professional care services, or it gives family care benefits to dependents and their families. 31 March 2017. From left to right: benefits (by type), total number of individual benefits granted, percentage of benefits granted in Castilla y León, and percentage of benefits granted within Spain.
